# Signaling, cancer cell plasticity, and intratumor heterogeneity

**DOI:** 10.1186/s12964-024-01643-5

**Published:** 2024-05-03

**Authors:** Marco Cordani, Ilaria Dando, Giulia Ambrosini, Pedro González-Menéndez

**Affiliations:** 1 Department of Biochemistry and Molecular Biology, Faculty of Biology, Complutense University, Madrid, 28040 Spain; 2 Instituto de Investigaciones Sanitarias San Carlos (IdISSC), Madrid, 28040 Spain; 3 Department of Neuroscience, Biomedicine and Movement Sciences, Biochemistry Section, University of Verona, Verona, 37134 Italy; 4 Departamento de Morfología y Biología Celular, School of Medicine, Julián Claveria 6, Oviedo, 33006 Spain; 5 Instituto Universitario de Oncología del Principado de Asturias (IUOPA), University of Oviedo, Oviedo, 33006 Spain; 6 Instituto de Investigación Sanitaria del Principado de Asturias (ISPA), Hospital Universitario Central de Asturias (HUCA), Oviedo, 33011 Spain

## Abstract

Cancer’s complexity is in part due to the presence of intratumor heterogeneity and the dynamic nature of cancer cell plasticity, which create substantial obstacles in effective cancer management. Variability within a tumor arises from the existence of diverse populations of cancer cells, impacting the progression, spread, and resistance to treatments. At the core of this variability is the concept of cellular plasticity - the intrinsic ability of cancer cells to alter their molecular and cellular identity in reaction to environmental and genetic changes. This adaptability is a cornerstone of cancer’s persistence and progression, making it a formidable target for treatments. Emerging studies have emphasized the critical role of such plasticity in fostering tumor diversity, which in turn influences the course of the disease and the effectiveness of therapeutic strategies. The transformative nature of cancer involves a network of signal transduction pathways, notably those that drive the epithelial-to-mesenchymal transition and metabolic remodeling, shaping the evolutionary path of cancer cells. Despite advancements, our understanding of the precise molecular machinations and signaling networks driving these changes is still evolving, underscoring the necessity for further research. This editorial presents a series entitled “Signaling Cancer Cell Plasticity and Intratumor Heterogeneity” in Cell Communication and Signaling, dedicated to unraveling these complex processes and proposing new avenues for therapeutic intervention.

## Introduction

The enigmatic and complex features of cancer pose significant obstacles for both researchers and healthcare professionals. Among these challenges, the issue of intratumor heterogeneity stands out as a particularly insurmountable obstacle. This phenomenon, often a precursor to cancer evolution and tumor progression, significantly hampers effective therapeutic strategies [1, 2]. Intratumor heterogeneity refers to the coexistence of genetically and phenotypically distinct cancer cell populations within a single tumor mass, with implications for tumor growth, metastasis, and therapeutic resistance [2]. For instance, genetic analysis of tumor samples from lung and kidney cancers has revealed considerable intratumor genetic heterogeneity, which is associated with increased resistance to targeted therapies [3, 4]. At the heart of intratumor heterogeneity lies cellular plasticity - the ability of cancer cells to undergo significant molecular and phenotypic alterations in response to a variety of stimuli, both genetic and epigenetic in origin [5, 6]. This ability allows cancer cells to adapt to their changing environment, including alterations in nutrient availability, hypoxia, immune response, and therapeutic intervention, among others [7]. Epigenetic changes, such as DNA methylation, histone modifications, and changes in non-coding RNAs, can lead to alterations in gene expression without changes in the underlying DNA sequence [8]. For example, hypermethylation of promoter regions can silence tumor suppressor genes, contributing to uncontrolled cell growth [9, 10]. Conversely, genetic changes, including point mutations, deletions, and chromosomal rearrangements, can lead to the activation of oncogenes or the inactivation of tumor suppressor genes, driving the development and progression of cancer [11, 12]. The plasticity of cancer cells, driven by these genetic and epigenetic changes, enables them to acquire new phenotypes that promote survival, growth, and progression, contributing to the heterogeneity observed within tumors [5] and between primary mass and metastases.

## The role of cellular plasticity in tumor progression

Recent research has highlighted the pivotal role of cellular plasticity in sustaining tumor heterogeneity, a key driver of cancer evolution, therapeutic response, and disease progression [5, 13, 14]. It has become increasingly clear that the interplay between distinct cellular states, a trademark of cellular plasticity, is instrumental in defining the clinical trajectory of various cancers. An excellent illustration of this plasticity is found in the flexible and dynamic nature of cancer cells, capable of switching between distinct stages of differentiation and a state of ‘stemming.’ This dynamic transformation is not a mere morphological change but rather encapsulates profound molecular and functional alterations, a process regulated by a complex network of intracellular signaling pathways and environmental cues [15–17]. A closer examination reveals a state of ‘stemness’ in these cancer cells, a phenomenon reminiscent of the properties found in embryonic or adult stem cells. This state, characterized by high cellular and metabolic plasticity [18, 19], imparts on these cancer cells, also known as cancer stem cells (CSCs), a unique set of features, which includes the capacity for self-renewal, potential for differentiation, and tumorigenic and metastatic properties [20–23]. One well-documented example is the aggressive triple-negative breast cancer (TNBC) subtype, wherein a fraction of the tumor cells exhibits a CSC phenotype. These cells have been found to display robust self-renewal capacity, potential for multilineage differentiation, and heightened resistance to chemotherapy, contributing significantly to tumor recurrence and patient relapse [24, 25]. Moreover, the versatile nature of CSCs endows them with another crucial attribute – invasiveness. This is exemplified in colorectal cancer, where CSCs have demonstrated an enhanced ability to invade surrounding tissues and form metastases, driving disease progression [26, 27]. The factors contributing to this invasiveness include an overactive Wnt/β-catenin pathway and epithelial-to-mesenchymal transition (EMT) inducers, both of which have been linked to cellular plasticity [28–30]. Understanding the intricate interplay of factors that regulate the switch between differentiation and stemness and the resultant phenotypic and functional changes in cancer cells can provide new inputs for therapeutic interventions to destabilize the plasticity and adaptability of cancer cells.

### The tumors’ key is reprogramming

Cancer cells undergo a transformative process skillfully directed by an intricate network of signaling transduction pathways. At the crux of this transformation lie two fundamental biological phenomena: the EMT and the metabolic shift from glycolysis to oxidative phosphorylation. These pivotal processes serve as central elements, guiding and shaping the evolutionary trajectory of cancer cells. The EMT pathway, initially identified as a fundamental developmental process, has been strategically exploited by cancer cells. It confers them a mesenchymal phenotype, enhancing their adaptability and conferring traits such as increased invasiveness and metastatic potential. Specific signaling pathways, such as TGF-β, Notch, and Wnt, are critical in initiating and maintaining the EMT [31–33]. In some contexts, these pathways trigger EMT by activating transcription factors such as Snail, Slug, Twist, or ZEB1/2, which in turn repress epithelial markers like E-cadherin, leading to loss of cell-cell adhesion and gain of migratory capacities [34, 35]. An exemplary case of this can be seen in non-small cell lung cancer (NSCLC), where the TGF-beta pathway has been implicated in driving EMT [31]. Concurrently, cancer cells undergo a dramatic metabolic reprogramming, shifting their energy production from glycolysis, favored under normal physiological conditions, to oxidative phosphorylation [18, 36, 37]. This metabolic shift is largely directed by signaling pathways such as PI3K/Akt/mTOR and AMPK, which profoundly influence the expression and activity of key metabolic enzymes [38–40]. For instance, glioblastoma cells have been shown to utilize oxidative phosphorylation instead of glycolysis, contradicting the classical Warburg effect, and the PI3K/Akt/mTOR pathway has been identified as a driving force behind this shift [40, 41]. These significant alterations do not occur in isolation. Rather, they are orchestrated through complex interactions between intracellular signaling pathways and extracellular cues, establishing an intricate network driving cancer cell plasticity [42, 43]. Examples of these cues include growth factors, extracellular matrix components, and immune cells present in the tumor microenvironment. These elements collaboratively modulate signaling pathways, dictating the behavior of cancer cells [44]. For instance, tumor-associated macrophages (TAMs) can promote EMT and metabolic reprogramming in neighboring cancer cells by secreting factors such as TGF-beta and IL-6 [45]. With this intricate web of events, the tumor microenvironment becomes an entity as dynamic and heterogeneous as the tumor itself, underscoring the need to delve further into the interplay between cancer cell plasticity and their surrounding milieu.

### Signaling, cancer cell plasticity, and intratumor heterogeneity

Despite the ability of cancer cells to acquire an oncogenic phenotype through intratumor heterogeneity and plasticity has been identified as a critical component of cancer progression and resistance to therapy, our understanding of the underlying molecular mechanisms and signaling pathways remains incomplete and presents a significant challenge to the effective treatment of cancer [46]. For example, cellular plasticity allows cancer cells to switch dynamically between different states of differentiation, which often promotes therapeutic resistance and metastasis. In some cases, cancer cells demonstrate phenotypic plasticity, adopting the properties of different cell types, such as mesenchymal or stem-like cells [46]. However, the exact signals that dictate these changes and the resulting phenotypic outcomes are still poorly understood. In other cases, metabolic plasticity, defined as the ability of cells to reprogram their metabolic pathways in response to both intrinsic and extrinsic factors, enables cancer cells to adapt to diverse and often hostile microenvironments [47, 48]. This metabolic flexibility contributes to survival, proliferation, invasion, and drug resistance. Yet, the intricate links between metabolic plasticity, cell signaling, and tumorigenesis are not entirely elucidated.

**Fig. 1:**
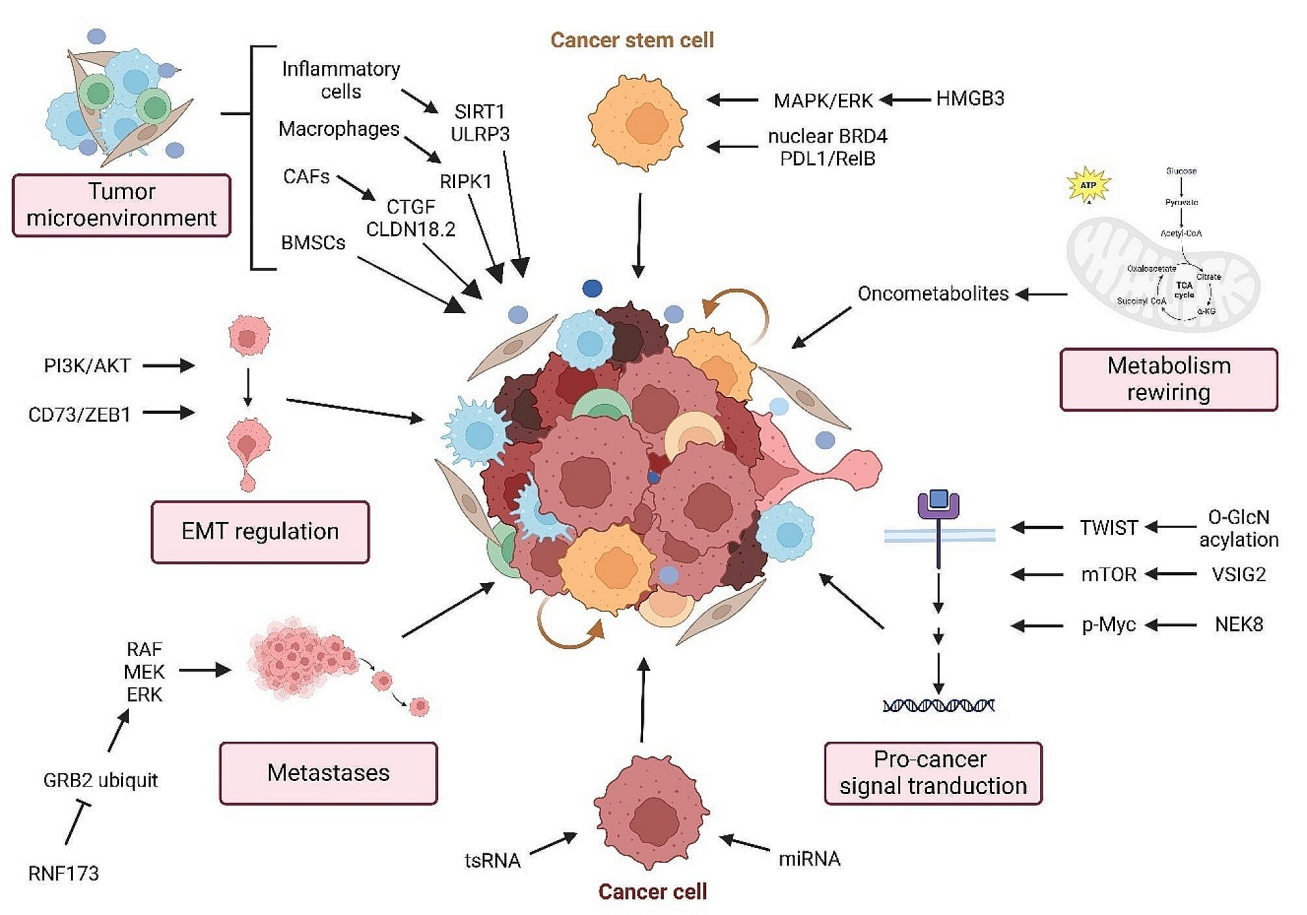
An integrative view of the molecular pathways and factors influencing cancer cell plasticity, the role of the tumor microenvironment, and the dynamic processes of EMT regulation, metastasis, and metabolism rewiring in cancer progression

Recognizing the urgency to bridge these knowledge gaps and bolster our understanding of tumor plasticity, Cell Communication and Signaling initiated this thematic series titled: “Signaling, Cancer Cell Plasticity, and Intratumor Heterogeneity”. This compilation of original research and review articles aims to dissect the convoluted mechanisms driving tumor heterogeneity and plasticity. Furthermore, it seeks to establish a dialogue on how our understanding of these processes can be leveraged to devise therapeutic strategies targeting these cellular transitions and adaptations. In essence, while we recognize the profound role of cellular plasticity in cancer, our understanding of its molecular features and the myriad signaling networks that dictate it remains a work in progress. By bringing together insights from eminent researchers and fostering collaborative discourse, this thematic series marks a significant stride in our quest to unravel the enigma of tumor heterogeneity and plasticity (Fig. 1).

The spectrum of articles within this special issue embodies cutting-edge research, elucidating the fundamental signaling pathways and networks that underpin tumor cell plasticity and intratumor heterogeneity. Herein, the reader will encounter a wealth of knowledge illuminating intricate processes, including Notch, Wnt, Myc, and Hedgehog signaling pathways that are instrumental in driving EMT and cancer stem cell properties. With an emphasis on the mechanisms of tumor progression, these articles shed light on the dynamics between tumor cells and their microenvironment, such as the paracrine signaling interactions that facilitate immune evasion and angiogenesis, hence fostering a fertile milieu for tumor proliferation and metastasis. One such illustrative example is the role of the transforming growth factor-beta (TGF-β) pathway, which modulates the tumor microenvironment and induces EMT, thereby promoting tumor progression [31, 49]. Each article weaves an intricate tale of how cancer cells maneuver their environment to enhance their survival and propagation.

A fundamental premise that steers this issue is the belief that a comprehensive understanding of the mechanisms implicated in cancer cell plasticity can herald novel therapeutic strategies. An in-depth investigation into the facets of CSCs and the mechanisms that drive tumor progression is vital to this end. Promising therapeutic approaches are discussed throughout this issue, such as targeted therapies against specific molecular aberrations in cancer cells or novel strategies to interfere with EMT and metabolic rewiring. For instance, one research article presents an innovative approach targeting the PI3K/AKT/mTOR pathway to counteract the plasticity and resilience of CSCs [50]. Another approach falls into the disruption of the Warburg effect, potentially starving the cancer cells of the energy necessary for their sustained growth and division [51].

### The cell plasticity and tumor heterogeneity in signaling collection

Our collection consists of 29 original research articles and literature reviews that we briefly described here as follows:

1. ***Elraglusib (formerly 9-ING-41) possesses potent anti-lymphoma properties, which cannot be attributed to GSK3 inhibition***. This study scrutinizes the efficacy of Elraglusib, a potential cancer treatment undergoing clinical trials, by analyzing its effects on different lymphoma cell lines and comparing its impact with other GSK3 inhibitors. The findings cast doubt on GSK3 being the primary target of Elraglusib in lymphoma, questioning the current understanding of the role of GSK3 as a therapeutic biomarker in NHL [52].
2. ***HMGB3 promotes the malignant phenotypes and stemness of epithelial ovarian cancer through the MAPK/ERK signaling pathway.*** The research sheds new light on the role of HMGB3 in the progression and metastasis of epithelial ovarian cancer, suggesting that targeting HMGB3, which influences the MAPK/ERK signaling pathway, could pave the way for innovative therapeutic approaches, potentially revolutionizing the treatment and improving the prognosis for women with ovarian cancer [53].
3. ***Intratumoural microbiota: from theory to clinical application.*** Ji and colleagues present a comprehensive review of the intriguing role of intratumoral microbiota in cancer progression and treatment, highlighting its potential in diagnostic, prognostic, and therapeutic applications. The review posits the microbiota as a potentially transformative avenue for cancer treatment, possibly fostering more effective and precise interventions [54].
4. ***Role of tRNA-derived small RNAs(tsRNAs) in the diagnosis and treatment of malignant tumours.*** This review highlights the emerging relevance of tsRNAs, small noncoding RNAs, in the tumorigenesis of various cancers, suggesting their potential as promising diagnostic markers and therapeutic targets. This insight could offer a novel approach to cancer treatment, presenting avenues for innovative research into the mechanisms of tumorigenesis and cancer progression [55].
5. ***PI3K/AKT signaling pathway as a critical regulator of epithelial-mesenchymal transition in colorectal tumor cells.*** Maharati et al. explore the critical role of the PI3K/AKT signaling pathway in the metastasis of colorectal cancer, particularly its influence on the epithelial-mesenchymal transition process. The study suggests that targeting this pathway might be a promising strategy to curtail colorectal cancer metastasis, possibly rewriting the narrative of patient prognosis and treatment [56].
6. ***RNF173 suppresses RAF/MEK/ERK signaling to regulate invasion and metastasis via GRB2 ubiquitination in Hepatocellular Carcinoma***. Zhou and team elucidate the role of RNF173 in hepatocellular carcinoma (HCC), demonstrating its impact on the progression and prognosis of the disease by inhibiting the RAF/MEK/ERK signaling pathway. The study highlights the potential of RNF173 as a novel prognostic molecule and therapeutic target, potentially offering a fresh perspective in pursuing more effective HCC treatments [57].
7. ***Retinoblastoma: present scenario and future challenges***. This comprehensive review discusses retinoblastoma’s genetic and molecular characteristics, emphasizing the potential of innovative and improved diagnostic and treatment strategies. The paper points to the critical role of early diagnosis and the exploration of genetic factors, heralding potentially groundbreaking advancements in managing this rare pediatric cancer [58].
8. ***BRD4/nuclear PD-L1/RelB circuit is involved in the stemness of breast cancer cells***. The research by Kim et al. unveils a groundbreaking axis of BRD4/nuclear PD-L1/RelB, intricately involved in the stemness of breast cancer cells, possibly offering a novel avenue for immunotherapies targeting intractable breast cancers. This research heralds a promising avenue, potentially revolutionizing the treatment landscape for breast cancer, where PD-L1 has emerged as a pivotal player in governing the stemness of breast cancer stem cells, thus opening up new perspectives for innovative therapies [59].
9. ***Characterization of gastric cancer-stimulated signaling pathways and function of CTGF in cancer-associated fibroblasts***. On the other hand, Choi et al. delve deep into the tumor microenvironment, specifically focusing on the pivotal role played by cancer-associated fibroblasts (CAFs) in gastric cancer progression. By meticulously dissecting the signaling pathways activated by CAFs and their subsequent effects on gastric cancer cells, this study brings to the fore the potential of targeting these fibroblasts therapeutically. Identifying the connective tissue growth factor (CTGF) as a central player in this interaction offers a promising avenue for novel therapies aimed at disrupting this crosstalk in the gastric cancer milieu [60].
10. ***Involvement of the SIRT1-NLRP3****p****athway in the****i****nflammatory****r****esponse***. The silent information regulator 2 homolog 1-NACHT, LRR and PYD domains-containing protein 3 (SIRT1-NLRP3) are critical in the inflammatory response. Chen and colleagues focus their review on the relationship of the SIRT1-NLRP3 pathway with melatonin, traumatic brain injury, neuroinflammation, depression, atherosclerosis, and liver damage [61].
11. ***Epigenetic meets metabolism: Novel vulnerabilities to fight cancer***. Scumaci and Zheng summarize in their review the crosstalk between Epigenetics and Metabolism in cancer, focusing on how metabolic reprogramming modifies the epigenome homeostasis by reacting with histone tails. In addition, they propose the use of drugs that target enzymes that preserve histone architecture [62].
12. ***The crescent-like Golgi ribbon is shaped by the Ajuba/PRMT5/Aurora-A complex-modified HURP.*** This article focuses on assembling the Golgi apparatus as a crescent-like ribbon structure. Chiu and colleagues have established that the Ajuba/PRMT5/Aurora-A complex unites the signals of protein methylation and phosphorylation to HURP, a common substrate of Aurora-A and PRMT5. Particularly, HURP P725 assembles the Golgi apparatus according to its shape [63].
13. ***Effect of tumor-associated macrophages on the pyroptosis of breast cancer tumor cells***. Pyroptosis induces a robust inflammatory response, which triggers cell death. Importantly, tumor cell pyroptosis can have a protective role in cancer by activating anti-tumor immunity. Ji et al. review how tumor-associated macrophages control pyroptosis in breast tumor cells and microenvironment [64].
14. ***SUMOylation of AnxA6 facilitates EGFR-PKCα complex formation to suppress epithelial cancer growth.*** The annexin A6 inhibits the phosphorylation of the epidermal growth factor receptor (EGFR) / extracellular signal-regulated kinase (ERK)1/2. However, this mechanism has not been extensively studied in cancer. Sheng et al. found that modification of the SUMOylation of Anx6 mediates cancer cell growth and the response to gefitinib treatment [65].
15. ***Fibroblast diversity and plasticity in the tumor microenvironment: roles in immunity and relevant therapies.*** CAFs play different roles in the tumor microenvironment. Therefore, CAFs have been considered a promising therapeutic target, but the current clinical trials are unsuccessful. Xu and colleagues summarize the heterogeneity of CAFs, focusing on their origin and activation. Furthermore, they discuss their role in tumor immunity and immunotherapies [66].
16. ***Castration promotes the browning of the prostate tumor microenvironment.*** Alvarez-Artime and colleagues describe for the first time that castration induces browning in the murine prostate tumor microenvironment. In particular, since the secretome of beige adipocytes diminishes the proliferation of androgen-responsive prostate cancer cells, browning could be considered a potential clinical strategy [67].
17. ***NEK8 regulates colorectal cancer progression via phosphorylating MYC.*** In this research article, Cao et al. illustrated that in colorectal cancer, the oncogene MYC, which is considered a tough protein to be hit due to its undruggable structure, could be targeted by taking advantage of the NEK8-driven phosphorylation on the Serine 405. Their findings highlight that a novel axis could be exploited to get rid of a crucial tumor-sustaining protein, as MYC is [68].
18. ***VSIG2 promotes malignant progression of pancreatic ductal adenocarcinoma by enhancing LAMTOR2-mediated mTOR activation.*** Xu and colleagues well described the importance of V-set and immunoglobulin domain containing 2 (VSIG2) in pancreatic ductal adenocarcinoma (PDAC) and in particular, its possible role in regulating proliferation, invasion, and metastasis. Indeed, they reported that high expression levels of this protein correlated with an advanced stage of the tumor; they demonstrated the direct link with the activation of mTOR and its related pathway, favoring the survival and aggressiveness of tumor cells. For this rationale, the authors propose VSIG2 as a possible new tumor marker for patients affected by PDAC and a conceivable target to limit tumor progression [69].
19. ***Comparative Immunological Landscape Between Pre- and Early-Stage LUAD Manifested as Ground-Glass Nodules Revealed by scRNA and scTCR Integrated Analysis***. In this article, the authors focused their attention on the malignant progression of precancer to early-stage lung adenocarcinoma by opening the way to the importance of the study of the processes that take place during the neoplastic formation. Through a single-cell analysis on primary tissues they showed that five principal modules have a key role during the progression, including cell proliferation, metabolism, immune response, mitochondria and cell adhesion [70].
20. ***Elucidating the molecular mechanisms underlying the induction of autophagy by antidepressant-like substances in C57BL/6J mouse testis model upon LPS challenge.*** Solek and colleagues describe the impact of anti-depressant-like substances (ALS) on mouse testis. While the administration of ALS induces apoptotic response via Golgi-mediated stress response, the coadministration of lipopolysaccharide promotes autophagy through ER stress [71]. .
21. ***Reciprocal regulation of TWIST1 and OGT determines the decitabine efficacy in MDS/AML.*** Chemoresistance in myelodysplastic syndromes (MDS) and acute myeloid leukemia (AML) is the main obstacle to therapeutical success. Li and colleagues propose O-GlcNAcylation as a stabilizer of the transcription factor TWIST by blocking its interaction with the ubiquitin E3 ligase CBLC. Therefore, this work opens the possibility of targeting O-GlcNAcylated proteins to reduce the number of MDS/AML relapses [72].
22. ***Claudin-18.2 mediated interaction of gastric Cancer cells and Cancer-associated fibroblasts drives tumor progression.*** This research study by Liu et al. highlights the critical role of Claudin-18.2 (CLDN18.2) in gastric cancer, particularly its involvement in mediating interactions between cancer cells and cancer-associated fibroblasts (CAFs). It provides valuable insights into how high CLDN18.2 expression correlates with advanced cancer stages and poor prognosis, potentially guiding future therapeutic strategies targeting CLDN18.2 in gastrointestinal tumors [73].
23. ***The Interplay between CD73 and ZEB1 in Papillary Thyroid Carcinoma***. Focusing on papillary thyroid carcinoma, this study by Vedovatto et al. delves into the relationship between CD73 and ZEB1, two significant markers linked to cancer malignancy and the epithelial-mesenchymal transition (EMT). The authors highlight the potential of targeting these molecules as therapeutic strategies, opening up new avenues for treating aggressive cancer behaviors [74].
24. ***MicroRNA Signatures Differentiate Types, Grades, and Stages of Breast Invasive Ductal Carcinoma (IDC): miRNA-target Interacting Signaling Pathways***. Dinesh-Kumar and colleagues identify distinct microRNA signatures that differentiate various types, grades, and stages of breast invasive ductal carcinoma (IDC), the most common form of breast cancer. They underscore the role of such microRNAs in cancer progression and their potential as biomarkers for early detection, offering promising new directions for diagnostics and treatment strategies [75].
25. ***Microvesicles from bone marrow-derived mesenchymal stem cells promote Helicobacter pylori-associated gastric cancer progression by transferring thrombospondin-2.*** Qi and colleagues previously described that bone marrow mesenchymal stem cells (BMSCs) promote *Helicobacter pylori*-associated gastric cancer progression via thrombospondin-2 secretion. Here, they found that this *Helicobacter pylori*-dependent stimulation is mediated by delivering trombospodin-2 in BMSC-derived microvesicles from different murine models [76], .
26. ***Caspase 6 promotes innate immune activation by functional crosstalk between RIPK1-IκBα axis in liver inflammation.*** Caspase 6 regulates innate immunity, and Lin and colleagues have studied the causal mechanism of this caspase in liver sterile inflammatory injury. The authors describe that caspase 6 is elevated in macrophages but not hepatocytes in ischemic livers. This effect is mediated by RIPK1-IκBα, which impacts NEK7/NLRP3 function in macrophages and hepatocyte ferroptosis [77].
27. ***Effects of a novel ANLN E841K mutation associated with SRNS on podocytes and its mechanism.*** Podocytes play a major role in causing Steroid-resistant nephrotic syndrome (SRNS), which is distinguished by proteinuria after glucocorticoid therapy. Lin et al. describe the effect of the pathogenic mutation ANLN E841K in SRNS patients in this work. This mutation activates podocytes’ PI3K/AKT/mTOR/apoptosis pathway [78].
28. ***Mechanisms underlying linear ubiquitination and implications in tumorigenesis and drug discovery.*** Li and colleagues summarize the most recent studies about linear ubiquitination. In linear ubiquitination, a substrate protein attaches to a head-to-tail polyubiquitin chain. The dysregulation of linear ubiquitination is related to cancer development. Thus, understanding the signaling pathways regulated by linear ubiquitination is critical to developing specific cancer treatment inhibitors [79].
29. ***Transcending Frontiers in Prostate Cancer: The Role of Oncometabolites on Epigenetic Regulation, CSCs, and Tumor Microenvironment to Identify New Therapeutic Strategies***. This review by Ambrosini et al. explores the complex role of oncometabolites in prostate cancer, focusing on their impact on epigenetic regulation, cancer stem cells, and the tumor microenvironment. It offers a comprehensive overview of current understandings and gaps, aiming to pave the way for novel therapeutic approaches targeting these metabolic aberrations [80].

### Call for papers

We cordially invite readers to immerse themselves in the papers of this special issue. We anticipate that it will serve as an insightful resource and an impetus for future investigations. It presents exciting avenues for therapeutic interventions, and it is our fervent hope that the knowledge disseminated herein will catalyze advancements that could profoundly alter the landscape of cancer treatment and enhance patient prognosis.
